# The therapeutic effects of *Lacticaseibacillus rhamnosus* on stress-induced anxiety: a systematic review of evidence from animal studies

**DOI:** 10.1017/gmb.2025.10015

**Published:** 2025-12-17

**Authors:** Iman Imtiyaz Ahmed Juvale, Alina Arulsamy

**Affiliations:** Neuropharmacology Research Laboratory, Monash University Malaysia, Jeffrey Cheah School of Medicine and Health Sciences, Malaysia

**Keywords:** anxiety, brain–gut axis, *Lacticaseibacillus Rhamnosus*, dysbiosis, immunomodulation, corticosterone, GABA

## Abstract

*Lacticaseibacillus rhamnosus* may modulate stress-induced anxiety, yet animal evidence has not been systematically evaluated. Following PRISMA guidelines, PubMed, Embase, and Scopus were searched (2011–2024) for animal studies evaluating the role of *L. rhamnosus* in stress-induced anxiety. Primary outcomes were behavioural anxiety measures; secondary outcomes included neuroendocrine, immune, epithelial, and microbiota changes. Fifteen studies met the inclusion criteria. Species included mice (n=7), rats (n=5), and hens (n=3). Stress models comprised chronic unpredictable mild stress (n=8), social defeat (n=2), maternal separation (n=1), restraint stress (n=1), and severe feather-pecking (n=3). Common strains were JB-1 (n=8), HN001 (n=2), LGG (n=2), LR-32 (n=1), 4B15 (n=1), and LR3201 (n=1). Of the 15 studies, 12 reported significant anxiolytic effects, most frequently in the elevated plus maze (7/10) and open-field test (6/9). JB-1 showed the most consistent behavioural improvement (7/8 studies). Mechanistic findings were reported in subsets of studies: HPA axis modulation in 4/15, monoamine changes in 4/15, GABAergic effects in 4/15, immune/anti-inflammatory changes in 4/15, tight junction restoration in 2/15, and gut microbiota or SCFA-related changes in 7/15. *L. rhamnosus*, particularly strain JB-1, shows consistent anxiolytic effects and multiple putative mechanistic pathways, though more rigorous and standardised preclinical designs are needed.

## Introduction

Stress is characterised as an uncontrollable and unpredictable situation that exceeds an individual’s ability to control and predict it (Moraes et al., 2022). The last few years have seen a notable surge in stress levels, marked as a significant societal burden. This increase in stress has been associated with the adoption of unhealthy lifestyle behaviours. Chronic elevation of stress levels can induce hyperactivity within the HPA axis, the primary neuroendocrine system involved in stress response, causing hypothalamic cells to secrete corticotropin-releasing hormone (CRH) and arginine vasopressin (AVP), stimulating the anterior pituitary to release adrenocorticotropic hormone (ACTH). Subsequently, ACTH can prompt the release of glucocorticosteroids such as cortisol, which exerts regulatory control over its synthesis and release via negative feedback loops (Tafet and Nemeroff, 2020). However, these negative feedback mechanisms are impaired during chronic stress, leading to the sustained activation of HPA axis (de Kloet et al., 2005; Tafet and Nemeroff, 2020). These profound alterations in HPA axis regulation, coupled with hypercortisolism, represent hallmark features associated with the development of anxiety disorders (Juruena et al., 2020; Piasecka et al., 2020).

Anxiety is a state of worry in response to a potential threat with an ambiguous or low probability of occurring (Watson et al., 2022). Alongside depression, it represents a significant proportion of the global disease burden, accounting for over 50% of disability-adjusted life years due to mental health disorders (Javaid et al., 2023). High anxiety and stress levels are risk factors for myocardial infarction and sudden cardiac arrest (Gustad et al., 2013; Manoj et al., 2018; Batelaan et al., 2022). Pharmacological interventions often target the stress–anxiety pathway, involving the upregulation of the HPA axis. Benzodiazepines, tricyclic antidepressants (TCAs), and selective serotonin reuptake inhibitors (SSRIs) are common medications (Tafet and Nemeroff, 2020), though they have adverse effects like cognitive impairment and drug dependence (Garakani et al., 2020; Chang et al., 2024). Anti-anxiety medication response rates vary, with significant recurrence rates, especially in generalised anxiety disorder (GAD) (Garakani et al., 2020). Despite debates about their efficacy and long-term risks, these medications remain primary treatments due to limited alternatives. Recent studies highlight the microbiota–gut–brain (MGB) axis’s role in mental health, with dysregulation contributing to anxiety disorders, emphasising the interplay between gut microbiota (GM) and mental health (Mörkl et al., 2020).

The gastrointestinal (GI) tract, which hosts a vast ecosystem of GM, plays crucial roles in human health, including immune modulation, vagal neurotransmission, tryptophan metabolism, neurotransmitter synthesis, endocrine function, and HPA axis regulation, influencing neuropsychiatric disorders (Evrensel et al., 2020; Huang and Wu, 2021; Butler et al., 2023). GM composition can be affected by stress, among other factors. Studies comparing GM in GAD patients and healthy controls reveal distinct microbial signatures, including reduced bacterial α-diversity in taxa like *Firmicutes* and *Tenericutes* and lower levels of specific bacteria such as *Eubacterium coprostanoligenes, Ruminococcaceae UCG-014*, and *Prevotella* 9 (Chen et al., 2019). Investigational treatments targeting GM, such as faecal microbiota transplantation, antibiotic therapy, prebiotics, and probiotics, show promise in alleviating anxiety by restoring GM balance (Savignac et al., 2016; Park et al., 2021; Baske et al., 2024). However, further elucidation of the specific bacterial taxa associated with healthy effects on anxiety and optimisation of strategies for integrating microbial-based interventions into clinical management paradigms are imperative areas of ongoing research.

*Lacticaseibacillus rhamnosus*, previously known as *Lactobacillus rhamnosus*, is an anaerobic, gram-positive, rod-shaped bacterium extensively utilised as a probiotic supplement owing to its notable therapeutic properties across various physiological systems (Suissa et al., 2023). Notably, its robust resistance to gastric acid and bile salts enables its survival and colonisation within the GI tract (de Champs et al., 2003; Mathipa-Mdakane and Thantsha, 2022), facilitated by its remarkable adhesive properties to the intestinal epithelial layer. This adhesive capacity serves a multifaceted role, impeding the adhesion and proliferation of exogenous pathogens while fostering intestinal integrity by forming protective biofilms, thus preserving cytoskeletal architecture (Forestier et al., 2001; Doron et al., 2005; Vélez et al., 2007; Marianelli et al., 2010; Zhang et al., 2011; Segers and Lebeer, 2014; Martín et al., 2019). Furthermore, supplementation with *L. rhamnosus* has demonstrated notable efficacy in ameliorating GI infections (Manzoni et al., 2006; Szajewska et al., 2007; Hojsak et al., 2010; Horvath et al., 2011; Szajewska et al., 2011; Boonma et al., 2014; Allonsius et al., 2017), exhibiting cytoprotective effects on intestinal epithelial cells (Seth et al., 2008; Wang et al., 2011), increasing resistance to pneumococcal infections, and eliciting immunomodulatory properties (Villena et al., 2014). Given the myriad multifaceted roles attributed to *L. rhamnosus* in physiological homoeostasis across various system functions and the burgeoning understanding of the MGB axis, *L. rhamnosus* has garnered significant attention in recent years for its potential therapeutic effects in psychiatric disorders. Thus, there is a growing need to investigate its potential therapeutic efficacy in stress-induced anxiety. Therefore, we aim to systematically review the existing literature to assess the therapeutic potential of *L. rhamnosus* in mitigating stress-induced anxiety, to synthesise evidence, highlight gaps, and offer direction for further investigation and future clinical application.

## Methodology

### Search strategies

The Preferred Reporting Items for Systematic Reviews and Meta-Analyses (PRISMA) guidelines were followed for this review (see Supplementary File 1). A comprehensive literature search was conducted in PubMed, EMBASE, and Scopus for studies published between 2011 and 2024, limited to English-language articles. The initial search used combinations of the terms “*Lacticaseibacillus rhamnosus*” AND “stress-induced anxiety” and “*Lactobacillus rhamnosus*” AND “stress-induced anxiety,” with a final search completed on 14 May 2024. Synonyms, strain identifiers, behavioural outcomes, stress paradigms, and controlled vocabulary were incorporated. The full Boolean strategy included: (“*Lacticaseibacillus rhamnosus*” OR “*Lactobacillus rhamnosus*” OR JB-1 OR HN001 OR LGG OR “LR-32” OR “ATCC 53103”) AND (anxiety OR anxiolytic OR “anxiety-like” OR “elevated plus maze” OR “open field” OR “light–dark” OR stress OR “social defeat” OR “maternal separation” OR restraint OR CUMS OR UCMS). MeSH/Emtree terms such as anxiety disorders, stress, psychological, and probiotics were also included. Subsequently, the identified studies were screened based on predetermined inclusion and exclusion criteria, as detailed below.

### Inclusion and exclusion criteria

The inclusion criteria for this review were as follows: (1) studies reporting behavioural and/or biochemical functional outcomes of *L. rhamnosus* in animal models of stress-induced anxiety in randomised and controlled studies; (2) inclusion of all species as identified in the literature; (3) inclusion of all sexes as identified in the literature; (4) inclusion of all doses and durations of *L. rhamnosus* supplementation; (5) all levels of stress-induced anxiety. The exclusion criteria were as follows: (1) studies involving bacterial strains other than *L. rhamnosus*; (2) studies addressing anxiety induced by other means (e.g. substance-induced anxiety); (3) *in vitro* studies; (4) studies lacking a separate control group; (5) non-original research articles such as systematic reviews, meta-analyses, editorials, letters, or abstracts; (6) duplicate publications; (7) studies without available full text. These inclusion and exclusion criteria were initially applied to the titles and abstracts of the identified studies, followed by a thorough review of the full texts of the remaining studies.

### Study selection and data extraction

The EndNote® software (Clarivate Analytics, Philadelphia, PA, USA) was employed during the initial stage of the search to identify and remove duplicate records. Two reviewers (I.I.A.J. and A.A.) independently screened the articles by title and abstract. Articles deemed relevant at this stage were subsequently reviewed in full text. Both reviewers performed data extraction independently, and disagreements were resolved through discussion. The primary outcome of interest was the “anti-anxiety effects,” defined as a reduction in stress–anxiety levels observed through behavioural tests in animal models and/or improvements in biochemical measurements. The extracted data included the following variables: first author’s surname and year of publication, sample size, species, intervention duration and dose, strain of *L. rhamnosus*, and a summary of the functional outcomes.

### Quality assessment

The quality of the included studies was assessed using the Systematic Review Centre for Laboratory Animal Experimentation (SYRCLE) Risk of Bias tool (Hooijmans et al., 2014), which evaluates the following domains: (1) sequence generation (selection bias), (2) baseline characteristics (selection bias), (3) allocation concealment (selection bias), (4) random housing (performance bias), (5) blinding (performance bias), (6) random outcome assessment (detection bias), (7) blinding (detection bias), (8) incomplete outcome data (attrition bias), (9) selective outcome reporting (reporting bias), and (10) other sources of bias. Each domain was rated as “+” (low risk of bias), “− ” (high risk of bias), or “?” (unclear risk of bias). Both reviewers independently conducted the quality assessment, and any discrepancies were resolved through discussion.

## Results

### Study selection

This review employed a structured narrative synthesis rather than a meta-analysis. All interpretations were based on qualitative patterns and thematic convergence, rather than numerical aggregation or statistical comparison of study outcomes. Although the number of studies reporting an effect is noted for context, these counts were not used to draw conclusions. The synthesis emphasises qualitative interpretation of behavioural, molecular, and microbiome outcomes. A total of 1226 studies were identified while searching the electronic databases: 458 from PubMed, 352 from Embase, and 416 from Scopus. Of these, 42 studies were excluded as duplicate records. After the initial screening of titles and abstracts, 784 studies were excluded as they were non-original research articles, including case reports, study protocols, editorials, systematic reviews, and meta-analyses. Then, 363 articles were further excluded as they did not meet the specific inclusion criteria for treatment. Of the remaining studies, 18 studies were excluded as they were without full text, leaving 15 original research articles. The process of study selection is shown in Supplementary Figure 1.

### Behavioural paradigms

Of the 15 animal studies in the systematic review, seven used mice, five used rats, and three used hens. Three studies used female animals. The included studies utilised various animal models to assess the therapeutic effects of *L. rhamnosus* across different stress intensities, encompassing mild, acute, and chronic stress paradigms. The most common type of disease model used was the unpredictable chronic mild stress (UCMS) model (n = 8), followed by the chronic social defeat model (n = 2), the early-life stress/maternal separation model (n = 1), and the restraint stress model (n = 1). The UCMS model induces mild to moderate stress through unpredictable stressors, such as light/dark cycle disruption and isolation, effectively replicating anhedonia, HPA axis dysregulation, and neuroimmune changes (Luo et al., 2025). In contrast, the chronic social defeat stress model induces moderate to severe stress by subjecting rodents to repeated social subordination, mirroring human stress-induced anxiety through social avoidance, HPA axis dysregulation, and neuroimmune alterations (Wang et al., 2021). The early-life stress/maternal separation model generates moderate to severe stress by separating neonates from their mothers for prolonged periods, disrupting HPA axis regulation, neurodevelopment, and emotional behaviour, thereby resembling early-life stress-induced anxiety in humans (Endo et al., 2021). Similarly, the restraint stress model induces moderate to severe stress by restricting an animal’s movement for fixed periods, effectively simulating human stress-induced anxiety through HPA axis activation, oxidative stress, and anxiety-like behaviours, reflecting both acute and chronic psychological stress effects (Xu et al., 2023). Additionally, some studies employed animals that had already exhibited stress-related behaviours, such as severe feather pecking in birds, which is characterised by chronic social stress and aggression. This model induces moderate to severe stress, as birds engage in excessive pecking directed at the feathers of conspecifics, leading to feather loss, skin damage, and increased corticosterone levels (Huang et al., 2023). The severe feather pecking model mimics key aspects of human stress-induced anxiety by replicating compulsive behaviours, social stress, and dysregulation of the HPA axis and neurotransmitter systems. Furthermore, this behaviour has been linked to GM alterations, reflecting gut–brain axis disturbances observed in human stress-related conditions.

### Bacterial strains and treatment protocols

The most common bacterial strain used was JB-1 (n = 8), followed by HN001 (n = 2), LGG (n = 2), LR-32 (n = 1), 4B15 (n = 1), and LR3201 (n = 1). The treatment was administered by either adding it to their food pellets (n = 1) or suspending them in a solvent (n = 14). Control groups were either given normal food pellets or the solvents (water, saline, or phosphate buffer saline) that were used to suspend the bacteria in the treatment group. The treatment period varied among studies, starting from 3 weeks and going up to 10 weeks. The treatments were provided daily (n = 10), a few days within a week (n = 3), or *ad libitum* (n = 2). The treatment dosage used ranged from 1 × 10^7^ CFU to 1 × 10^10^ CFU. A summary of the screened and extracted data utilised in this review is provided in Table 1.

**Table 1.** A summary of significant findings of *L. rhamnosus* treatment in relation to stress-induced anxiety based on animal studies (table tabulated in order of discovery/publication year). Gamma-aminobutyric acid-type A receptor (GABA_A_R); messenger ribonucleic acid (mRNA); interleukin-10 (IL-10); gut microbiota (GM); corticotropin-releasing hormone receptor-1 (CRHR1); total N-acetylaspartate (tNAA); G protein-coupled receptor 43 (GPR43); dopamine receptor D2 (DRD2); 5-hydroxytryptamine receptor (5-HTR); N-methyl-D-aspartate receptor (NMDAR); chemokine (C-C motif) ligand 2 (CCL2); tumour necrosis factor-alpha (TNF-α); metabotropic glutamate receptor (Grm4); Zonula Occludens-1 (ZO-1); transfer ribonucleic acid (tRNA)

References	Sample size (sex)	Animal species	Strain of *L. rhamnosus*	Intervention duration (dosage)	Significant findings
Bravo et al. (2011)	36 (male)	BALB/c mice	JB–1	4 weeks daily; (5 × 10^9^ CFU/ml)	JB–1 significantly reduced anxiety-like behaviour, as shown by an increase in the number of entries into the open arms of the elevated plus maze (t = 4.662, df = 34; P < 0.001) and a reduction in immobility time in the forced swim test (t = 3.926, df = 14; P < 0.01) compared to the control group. Neurochemical analysis revealed a significant upregulation of GABAB1b receptor mRNA levels in the cingulate cortex 1 and prelimbic cortical areas (t = 3.485, df = 10, P < 0.01) and GABA_A_α2 receptor mRNA levels in the dentate gyrus (t = 5.967, df = 10, P < 0.001), compared to the control. Plasma corticosterone levels were significantly lower in the treated group [F (1, 28) = 11.409; P = 0.0022] compared to the control [F (1, 28) = 73.90; P < 0.0001], indicating a reduced stress response.
Bharwani et al. (2017)	43 (male)	C57BL/6 mice	JB–1	20 times over a period of 4 weeks; 200 μl (1.67 × 10^9^ CFU/ml)	JB–1 significantly attenuated deficits in rearing and improved exploration in the open-field test [F (1, 14) = 6.888; P = 0.02], while also increasing the frequency of entry into the light compartment in the light–dark box test [F (1, 57) = 5.171; P = 0.027, post hoc, P < 0.05], compared to the control. JB–1 decreased stress-related activation of dendritic cells [F (1, 15) = 8.224; P = 0.012; post hoc, P < 0.05] and significantly increased IL–10+ regulatory T cells [F (1, 16) = 5.621; P = 0.031) compared to the control. JB–1 treatment did not prevent stress-induced gut microbiota changes, but control groups treated with JB–1 exhibited alterations in 75 metabolites, with known immunomodulatory and neuroactive properties. Tyramine levels were the only metabolite significantly stabilised by JB–1 treatment (P < 0.05).
Neufeld et al. (2019)	28 (male)	Sprague Dawley rats	LGG	8 weeks daily (1 × 10^8^ CFU/ml)	LGG significantly reduced anxiety-like behaviours in stressed animals, as shown by an increase in the distance travelled in the open-field test (P = 0.01) compared to the stressed control. The addition of prebiotics to LGG supplementation further enhanced the anxiolytic effects compared to LGG alone (P = 0.008). LGG normalised aberrant mRNA expression of glucocorticoid receptors (P < 0.0001) and significantly upregulated CRHR1 mRNA expression (P < 0.001) compared to untreated stressed animals.
Kochalska et al. (2020)	48 (male)	Wistar rats	JB–1	4 weeks daily; 200 μl (~1.7 × 10^9^ CFU)	JB–1 significantly reduced anxiety-like behaviour in the elevated plus maze test, as indicated by an increase in time spent in the open arms and a reduction in freezing behaviour compared to controls (P < 0.003). JB–1 treatment increased the levels of glutamine + glutathione (~14%; P < 0.05) and tNAA (8%; P < 0.05), while restoring the levels of GABA, glutamate, glutamate + glutamine, and total creatine that were altered due to stress, as measured by magnetic resonance spectroscopy.
Oh et al. (2020)	40 (male)	C57BL/6 mice	4B15	10 weeks *ad libitum*; (1 × 10^7^ CFU/ml)	4B15 significantly reduced anxiety-like behaviours in stressed rats, as indicated by an increase in time spent in the centre of the open-field test (P < 0.005) and an increase in open-arm exploration in the elevated plus maze (P < 0.001) compared to stressed controls. 4B15 supplementation ameliorated stress-induced gut dysbiosis, restoring microbial diversity and increasing beneficial bacterial taxa compared to untreated stressed rats. 4B15 significantly decreased serum corticosterone levels (P < 0.001), and mRNA and protein expression levels of CRHR1 (P < 0.05) and NMDAR subunit 2 (P < 0.001) as compared to the control. 4B15 treatment increased serotonin (P < 0.001) and tryptophan hydroxylase–1 protein levels (P < 0.05). Additionally, mRNA and protein expression levels of GPR43 (P < 0.05), DRD2 (P < 0.05), and 5-HTR1A (P < 0.005) were upregulated compared to stressed controls.
Liu et al. (2020)	48 (male)	C57BL/6 mice	JB–1	4 weeks daily; 200 μl (1 × 10^9^ CFU)	JB–1 treatment did not significantly affect anxiety-like behaviours in stressed animals, as indicated by no significant changes in time spent in the centre of the open-field test, open arm exploration in the elevated plus maze, or light compartment preference in the light–dark test compared to stressed controls. JB–1-treated stressed animals exhibited reduced hippocampal CRHR1 mRNA expression compared to untreated stressed animals (0.37 ± 0.049, g = 2.32, P = 0.0020). JB–1 treatment significantly lowered Ly6Chi monocyte expression (5.73 ± 0.62%, g = 1.46, P = 0.0238) and monocyte chemoattractant CCL2 expression levels compared to the control group (0.284 ± 0.034, g = 1.02, P = 0.0260).
Mindus et al. (2021a)	86 (female)	Non-beak-trimmed White Leghorn laying hens	JB–1	On weekdays for 9 weeks; (5 × 10^9^ CFU/ml)	The JB–1 group exhibited a significant 2.5-fold reduction in feather-pecking behaviour compared to the control group (P < 0.05). JB–1 treatment prevented stress-induced gut dysbiosis, maintaining microbial diversity and increasing beneficial bacterial taxa (median: 0.590) compared to stressed controls (median: 0.619; P < 0.05). Regulatory T-cell levels were significantly elevated in the caecal tonsils (P < 0.001) and spleen (P = 0.088) compared to the control group.
Liu et al., 2021a,b	46 (male)	BALB/c mice	JB–1	4 weeks daily; 3 ml (1 × 10^9^ CFU/ml)	JB–1 supplementation significantly reduced anxiety-like behaviour in sham-operated stressed animals, as shown by an increase in time spent in the light compartment of the light–dark test (183.5 ± 24.2 s, g = 1.3779, P = 0.0048) and an increase in open-arm exploration in the elevated plus maze (17.8 ± 2.7 s, g = 1.0509, P = 0.0275). However, these anxiolytic effects were absent in vagotomised animals, suggesting a vagus nerve-dependent mechanism. JB–1-treated sham animals exhibited lower corticosterone levels (12.437 ± 1.695 ng/ml, g = 1.9237, P = 0.0054) compared to untreated stressed controls. JB–1 treatment increased splenic CD4^+^CD25^+^Foxp3^+^ T-cell expression levels (24.3 ± 0.8%, g = 2.2269, P = 0.0134) and reduced hippocampal microglia expression levels (0.795 ± 0.0036, g = 2.0363, P = 0.0351) compared to stressed controls.
Mindus et al. (2021b)	354 (female)	Non-beak-trimmed White Leghorn laying hens	JB–1	On weekdays for 9 weeks (5× 10^9^ CFU)	JB–1 supplementation had no significant effect on feather-pecking behaviour, with no observable differences in pecking incidents between treated and control groups. JB–1 supplementation reversed stress-induced immunological and metabolic changes, leading to an increase in splenic cytotoxic T cells [22.5 ± 0.56; F (1, 23) = 31.92, P < 0.0001], splenic T helper cells [29 ± 1.2; F (1, 23) = 6.30, P = 0.020], splenic regulatory T cells [19 ± 1.1; F (1, 23) = 18.72, P < 0.001], caecal tonsil regulatory T cells [13.0 ± 0.75; F (1, 23) = 8.22, P = 0.009], and tryptophan levels (8%; P < 0.001) compared to stressed untreated hens.
Huang et al. (2022)	59 (male)	Sprague Dawley rats	HN001	6 weeks daily; 1 ml (1 × 10^9^ CFU/ml)	HN001 significantly reduced anxiety-like behaviour in stressed rats, as indicated by an increase in time spent in the centre of the open-field test (P < 0.05) and an increase in open-arm exploration in the elevated plus maze (P < 0.05) compared to stressed controls. HN001 treatment corrected stress-induced gut microbiota alterations, restoring microbial diversity and increasing beneficial bacterial taxa compared to untreated stressed animals. HN001 significantly reduced systemic and neuroinflammatory markers of serum, brain, and colon IL–6, TNF-α, IL–1β, and IL–18, compared to stressed controls (P < 0.05). HN001 effectively reversed stress-induced reductions in monoamine neurotransmitters, leading to an increase in serotonin, dopamine, and norepinephrine levels in both serum and brain tissue (P < 0.05).
Chudzik et al. (2022)	20 (male)	Wistar rats	JB–1	8 weeks daily; 0.2 ml (1.7 × 10^9^ CFU)	JB–1 significantly reduced anxiety-like behaviour in stressed animals, as indicated by an increase in open-arm exploration in the elevated plus maze test compared to the control group (P < 0.05). JB–1 supplementation significantly increased hippocampal glutamine and glutathione concentrations compared to the control group (P < 0.05). JB–1 stabilised the levels of most gut-derived metabolites, maintaining levels of non-stressed controls, except for taurine, which remained significantly altered.
Faucher et al. (2022)	144 (male)	C57BL/6 mice	LGG	3 weeks daily; 0.12 mg (2.4 × 10^7^ CFU)	The active form of LGG significantly reduced anxiety-like behaviour, as indicated by an increase in time spent in the centre of the open-field test and a reduction in freezing behaviour compared to the control group (P < 0.03). However, the inactive form of LGG did not produce similar anxiolytic effects, showing no significant differences from controls.
Dalziel et al. (2023)	63 (male)	Wistar Kyoto rats	HN001	5 weeks *ad libitum*; (1 × 10^9^ CFU)	No significant behavioural differences were observed between the HN001-treated and control groups, as measured by the open-field test and elevated plus maze performance. Gene expression analysis revealed a twofold increase in GRM4 expression in the amygdala of HN001-treated animals compared to controls (P < 0.05). HN001 treatment significantly altered gut microbiota composition, leading to an increase in *Enterobacteriaceae* abundance compared to controls (FDR = 0.002). Functional gene profiling showed upregulation in genes associated with cell envelope formation (FDR = 0.0001–0.0242), amino acid (FDR = 0.0393) and carbohydrate metabolism (FDR = 0.0369–0.0485), iron acquisition (FDR = 0.0167–0.0303), and microbial stress response (FDR = 0.0014–0.0167) following HN001 treatment.
Song et al. (2023)	- (male)	C57BL/6 mice	L3201	3 weeks daily; (1 × 10^9^ CFU)	L3201 significantly reduced anxiety-like behaviour in stressed animals, as indicated by an increase in open-arm exploration in the elevated plus maze test compared to the stressed control group (P < 0.05). L3201 treatment significantly elevated the expression levels of specific faecal metabolites, such as those associated with aminoacyl-tRNA biosynthesis and those linked to valine, leucine, and isoleucine biosynthesis pathways in stressed mice compared to controls.
Huang et al. (2023)	216 (female)	Hy-Line Brown pullets	LR–32	8 weeks daily; (1 × 10^10^ CFU)	LR–32 administration significantly attenuated stress-induced reductions in egg production, restoring levels to baseline compared to stressed untreated hens (P < 0.05). LR–32-treated hens exhibited a significant reduction (one-fold lower) in feather-pecking behaviour, compared to stressed controls. LR–32 intervention reversed gut dysbiosis in stressed hens, increasing the abundance of beneficial microbial populations. This microbial restoration was associated with an upregulation of mRNA and protein abundance of ZO–1 (P = 0.012) and occludin (P = 0.023), suggesting improved gut-barrier integrity. LR–32 treatment significantly enhanced serotonin metabolism, with the upregulation of genes involved in serotonin synthesis (P < 0.05) and increased butyric acid levels compared to stressed controls (P < 0.01).

### Study quality

A risk-of-bias assessment using SYRCLE’s tool revealed variable methodological reporting across the 15 included studies. Only four studies clearly described random sequence generation, while the remaining eleven did not provide sufficient detail. All studies reported comparable baseline characteristics across groups. Allocation concealment was explicitly described in six studies, with nine rated as unclear due to the absence of methodological information. Randomisation into experimental versus control groups was reported in eleven studies, although most did not elaborate on the method used. Blinding of personnel and outcome assessment were mentioned in seven studies, whereas the others lacked the necessary information to judge detection or performance bias. Incomplete outcome data and selective reporting could not be confidently judged as low risk across all studies. Although no obvious omissions were identified, approximately one-third of the studies did not provide enough detail to rule out attrition or reporting bias. Other potential sources of bias were also inconsistently reported, with ten studies stating that they had no conflicts of interest, two declaring conflicts, and three providing no statement. Taken together, the predominance of unclear ratings, particularly for sequence generation, allocation concealment, blinding, and outcome reporting, indicates that the overall certainty of the evidence is low to moderate. Consistency in baseline characteristics and generally complete datasets strengthens confidence to some extent; however, the limited methodological transparency typical of preclinical studies reduces the robustness of the conclusions and should be considered when interpreting the findings (Supplementary File 2).

### The effects of L. Rhamnosus on stress-induced anxiety in animals

#### Behavioural assessment

**Figure 1.**
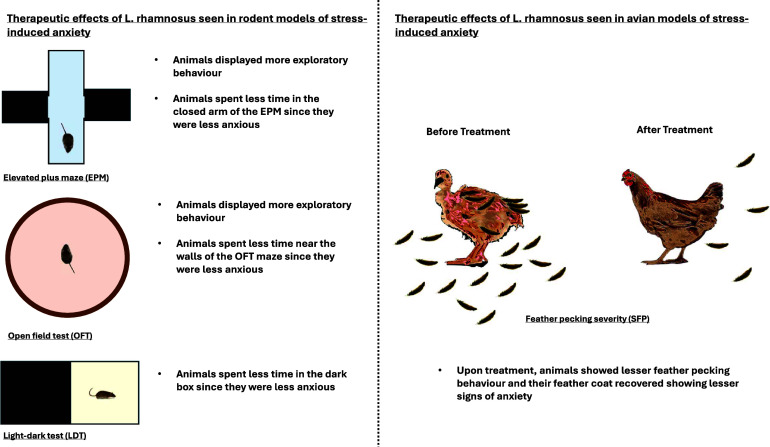
Summary diagram of the effects of L. rhamnosus on anxiety-like behaviour.

In our review of fifteen studies, twelve demonstrated significantly lower anxiety levels in stressed animals treated with *L. rhamnosus* compared to those receiving a placebo (Table 2). The remaining three studies did not observe significant behavioural changes. The predominant methods used to assess anxiety levels were the elevated plus maze (EPM; n = 10) and the open-field test (OFT; n = 9). Additionally, three studies employed the light–dark test (LDT). In studies involving hens (n = 3), feather-pecking severity (SFP) was used to evaluate stress-induced anxiety. Seven out of ten studies using the EPM observed a significant reduction in anxiety levels in treated animals, indicated by increased entries into the open arms and a higher percentage of time spent in the open arms (Bravo et al., 2011; Kochalska et al., 2020; Oh et al., 2020; Liu et al., 2021b; Chudzik et al., 2022; Huang et al., 2022; Song et al., 2023). Animals in the treatment group appeared calmer and more relaxed compared to controls (Kochalska et al., 2020). In the OFT, six out of nine studies reported a significant reduction in anxiety, characterised by increased exploratory behaviour and reduced thigmotaxis (time spent near the walls of the maze) (Bharwani et al., 2017; Neufeld et al., 2019; Oh et al., 2020; Liu et al., 2021b; Faucher et al., 2022; Huang et al., 2022). Two out of three studies using the LDT noted lower anxiety levels in treated animals, evidenced by increased time spent exploring the light compartment (Bharwani et al., 2017; Liu et al., 2021b). Similarly, two out of three studies on hens reported a significant reduction in SFP behaviour in those treated with *L. rhamnosus* (Huang et al., 2023; Mindus et al., 2021a). One study found a onefold reduction in SFP frequency (Mindus et al., 2021a), while another reported a 2.5-fold decrease compared to the placebo group (Huang et al., 2023). These studies also noted improved feather cover and the occurrence of gentle feather pecking behaviour, which is important for establishing positive social relationships among chicks (Mindus et al., 2021b). Additionally, hens with stress-induced anxiety experienced lower egg production, which returned to healthy levels following *L. rhamnosus* treatment (Huang et al., 2023) (Figure 1).

**Table 2.** Summary of *L. rhamnosus* strains and related models showing effects on stress-induced anxiety

Strain	Animal model	Behavioural effect	Effect size
JB–1	BALB/c mice	↓ Anxiety (EPM, FST)	t = 4.66 (EPM), t = 3.93 (FST)
JB–1	C57BL/6 mice	↓ Anxiety (OFT, LDB)	F (1,14) = 6.89 (OFT), F (1,57) = 5.17 (LDB)
LGG	Sprague Dawley rats	↓ Anxiety (OFT distance)	P = 0.01
JB–1	Wistar rats	↓ Anxiety (EPM)	P < 0.003
4B15	C57BL/6 mice	↓ Anxiety (OFT, EPM)	P < 0.005/P < 0.001
JB–1	C57BL/6 mice	↔ Anxiety	No significant change
JB–1	White Leghorn hens	↓ Feather-pecking	2.5-fold reduction, P < 0.05
JB–1	BALB/c mice (sham)	↓ Anxiety	Light–dark: g = 1.38, P = 0.0048; EPM: g = 1.05, P = 0.0275
HN001	Sprague Dawley rats	↓ Anxiety (OFT, EPM)	P < 0.05
HN001	Wistar Kyoto rats	↔ Anxiety	No significant change
L3201	C57BL/6 mice	↓ Anxiety (EPM)	P < 0.05
LR–32	Hy-Line Brown pullets	↓ Feather-pecking	1-fold reduction, P < 0.05

*Note*:↓ = reduced anxiety/stress behaviour; ↔ = no significant behavioural change; EPM = elevated plus maze; OFT = open-field test; LDB = light–dark box; P < 0.05 = statistically significant

#### Biochemical assessment: Neurotransmitters, receptors, and the HPA axis

Of the 15 studies, 14 reported significant biochemical changes in treated animals compared to the control and placebo groups. The one study that did not report such changes did not conduct any biochemical assessment. Stressed animals exhibited elevated levels of corticosterone. Treatment with *L. rhamnosus* significantly reduced serum levels of corticosterone, CRH, glucocorticoid and mineralocorticoid receptors, as well as their mRNA and protein expression levels in four studies (Bravo et al., 2011; Liu et al., 2020; Oh et al., 2020; Liu et al., 2021b). Additionally, stressed animals showed significantly lower serum and brain expression levels of dopamine, norepinephrine, serotonin, tryptophan, and tryptophan hydroxylase-1 and 2 (TPH1 and TPH2) in four studies (Huang et al., 2022, 2023; Oh et al., 2020; Mindus et al., 2021b). *L. rhamnosus* treatment reversed these expression levels. In stressed animals, serotonin and dopamine receptor expression levels were reduced by 0.6-fold and 0.2-fold, respectively, but treatment restored these to control levels (Oh et al., 2020).

Moreover, four studies reported that stressed animals had altered expression levels of gamma-aminobutyric acid (GABA) and its receptors (GABA_A_α2 and GABA_B_1b) (Bravo et al., 2011; Kochalska et al., 2020; Liu et al., 2020; Chudzik et al., 2022). Lower expression levels of GABA in stressed animals were reversed by *L. rhamnosus* treatment (Kochalska et al., 2020; Chudzik et al., 2022). Treated animals exhibited higher expression of GABA_B_1b messenger ribonucleic acid (mRNA) in the cingulate cortex 1 and the prelimbic cortical regions and lower expression levels in the basolateral amygdala, central amygdala, locus coeruleus, dentate gyrus, CA1, and CA3 compared to the placebo group (Bravo et al., 2011). In contrast, treated animals had lower expression levels of GABA_A_α2 mRNA in the cingulate cortex 1, prelimbic and infralimbic cortical regions, and the basolateral and central amygdala compared to the placebo group. The dentate gyrus of treated animals showed higher levels of GABA_A_α2 mRNA compared to the placebo group (Bravo et al., 2011), while another study noted lower expression levels of GABA_A_α2 mRNA in the hippocampus (Liu et al., 2020).

Stress significantly downregulated the expression levels of G-protein-coupled receptor 43 (GPR43), a modulator of short-chain fatty acids with a neuroprotective role (Oh et al., 2020). These levels were normalised upon treatment. Two studies noted that stressed animals exhibited significantly lower levels of glutamate, glutathione, glutamine, total creatine, total choline, N-acetylaspartate (NAA), total N-acetylaspartate (tNAA), and glutamate metabotropic receptor-4 (GRM4) (Kochalska et al., 2020; Dalziel et al., 2023). *L. rhamnosus* treatment restored these neurochemical levels to those observed in the control group.

#### Biochemical assessment: Immune mediators and inflammation

*L. rhamnosus* has demonstrated significant immunomodulatory effects in various studies. Four studies noted that treated animals exhibited a notable increase in cytotoxic T cells, T helper cells, and a cluster of differentiation antigen 4 positive (CD4+)CD25+ and interleukin-10 (IL-10)-expressing regulatory T (TREG) cells (Bharwani et al., 2017; Liu et al., 2021b; Mindus et al., 2021a). Conversely, expression levels of Ly6C^hi^ monocytes, the monocyte chemoattractant chemokine (C–C motif) ligand-2 (CCL2), major histocompatibility complex class II positive (MHCII+) CD11c + and CD80-expressing dendritic cells, and hippocampal microglia were significantly reduced in treated animals compared to the stress-induced group (Bharwani et al., 2017; Liu et al., 2020, 2021b). Furthermore, stressed animals showed elevated levels of serum, brain, and colon pro-inflammatory markers, including IL-6, tumour necrosis factor-alpha (TNF-α), IL-1β, IL-18, Toll-like receptor 4 (TLR4), inducible nitric oxide synthase (iNOS), and cyclooxygenase-2 (COX-2) (Bharwani et al., 2017; Oh et al., 2020; Huang et al., 2022; Huang et al., 2023). Treatment with *L. rhamnosus* significantly suppressed these levels, with a positive correlation observed between brain and colon inflammation markers. Brain-derived neurotrophic factor (BDNF) levels were also significantly higher in the treatment group compared to the placebo group (Oh et al., 2020). Treated animals displayed lower levels of apoptotic proteins such as B cell lymphoma-type 2 (BCL-2), Bcl-2-associated X protein (BAX), and caspase-3 than stressed animals (Oh et al., 2020).

#### Biochemical assessment: Epithelial barrier integrity

Two studies further noted that stressed animals had reduced expression of tight junction proteins, including ZO-1, occludin, claudin-1, and claudin-5, in both brain and intestinal tissues. All these levels, except claudin-1, were restored by *L. rhamnosus* treatment (Oh et al., 2020; Huang et al., 2023). Intestinal epithelial permeability, indicated by 4-kDa fluorescein isothiocyanate-dextran (FD4), was significantly higher in stressed animals but was reduced in the treated group (Oh et al., 2020). Histological analysis revealed that stressed animals had less firm intestinal epithelial cells and damaged intestinal villi with thin intestinal mucosa; however, pre-treatment with *L. rhamnosus* prevented these stress-induced changes (Huang et al., 2022). Examination of neuronal morphology showed a higher number of Nissl bodies in the control and treatment groups compared to the stressed group.

#### Biochemical assessment: Microbiota composition and microbial-derived metabolites

Furthermore, seven studies reported that *L. rhamnosus* treatment corrected stress-induced dysbiosis of the GM and levels of gut-derived metabolites (Oh et al., 2020; Mindus et al., 2021b; Chudzik et al., 2022; Huang et al., 2022; Dalziel et al., 2023; Huang et al., 2023; Song et al., 2023). In hens, treatment significantly increased the abundance of taxa commonly associated with health in prior studies such as *Firmicutes, Faecalibacterium, Dialister, Succinatimonas*, and *Lactobacillus*, while reducing taxa commonly associated with adversity in prior studies like *Proteobacteria, Desulfovibrio*, and *Prevotella.* Positive correlations were observed between *Faecalibacterium, Lactobacillus, and Succinatimonas* with serum concentrations of tryptophan and serotonin, as well as TPH2 and occludin gene expression (Huang et al., 2023). Increased abundance of *Lactobacillus* was associated with reduced SFP behaviour, while *Dialister* correlated positively with intestinal and brain ZO-1 gene expression levels. In contrast, *Desulfovibrio* and *Prevotella* were positively correlated with SFP behaviour and increased levels of TNF-α and TLR4, but negatively correlated with tryptophan, serotonin, butyric acid levels, and TPH2, ZO-1, and occludin gene expression. In rodents, stressed animals exhibited higher abundance of *Proteobacteria, Lachnospiraceae, Staphylococcaceae, Bacteroides, Caproiciproducens, Clostridium, Desulfovibrio, Turicibacter, Enterococcus*, and *Helicobacter*, which were reversed by *L. rhamnosus* treatment. Higher abundance of *Clostridium, Enterococcus, Caproiciproducens*, and *Desulfovibrio* correlated positively with increased anxiety-like behaviours, and elevated expression of pro-apoptotic proteins and inflammatory markers (Oh et al., 2020).

**Figure 2.**
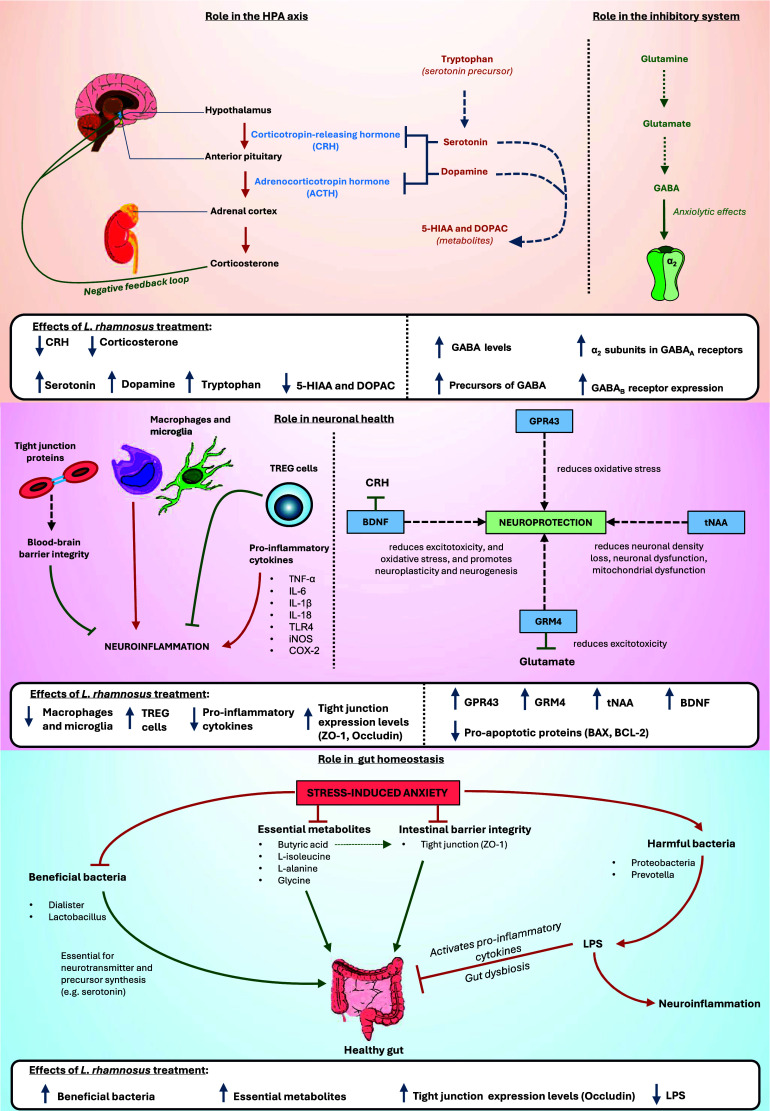
Summary diagram of biochemical pathways affected by L. rhamnosus treatment instress-induced anxiety. 5-Hydroxyindoleacetic acid (5-HIAA) 3,4-Dihydroxyphenylacetic acid(DOPAC); gamma-aminobutyric acid (GABA); Regulatory T-cells (TREG cells); Tumour NecrosisFactor alpha (TNF alpha); Interleukin 6 (IL-6); Toll-like receptor 4 (TLR4); inducible nitric oxidesynthase (iNOS); Cyclooxygenase-2 (COX-2); G-protein coupled receptor 43 (GPR43); Brain-derivedneurotrophic factor (BDNF); total N-acetylaspartate (tNAA); Glutamate Metabotropic Receptor 4(GRM4); Zonula occludens-1 (ZO-1); Lipopolysaccharides (LPS).

Stressed animals exhibited elevated levels of lipopolysaccharides (LPS), 5-hydroxyindoleacetic acid (5-HIAA), and 3,4-dihydroxyphenylacetic acid (DOPAC); however, administration of *L. rhamnosus* normalised these levels (Huang et al., 2022, 2023). Conversely, stressed hens had lower levels of propionic acid and butyric acid. While *L. rhamnosus* treatment effectively restored butyric acid levels, it did not significantly alter propionic acid levels (Huang et al., 2023). In rodent models, stressed animals demonstrated reduced expression levels of several metabolites, including L-alanine, L-isoleucine, cholesterol, D-galactose, α-L-galactofuranoside, 4-methylmandelic acid, glycine, 2-ethylhexanoic acid, 3-oxaoct-4-en-2-imine, and propanoic acid (Song et al., 2023). Treatment with *L. rhamnosus* significantly increased the levels of L-alanine, L-isoleucine, D-galactose, α-L-galactofuranoside, 4-methylmandelic acid, glycine, 2-ethylhexanoic acid, 3-oxaoct-4-en-2-imine, and propanoic acid, effectively reversing the stress-induced reductions (Bharwani et al., 2017; Song et al., 2023). Conversely, stressed animals showed higher L-threose and α-tocopherol expression levels, which were downregulated following *L. rhamnosus* administration (Song et al., 2023) (Figure 2).

## Discussion

Based on the literature findings from our review, we identified compelling evidence that *L. rhamnosus* exhibits significant anxiolytic effects in animal models. Significant biochemical alterations were still reported even in studies where behavioural changes were not observed. Given the multifaceted nature of stress-induced anxiety, discrepancies in behavioural outcomes are to be expected. For instance, some studies reported behavioural changes using the EPM but not the OFT. This is understandable, as these behavioural tests assess different levels of anxiety; what might not be detectable in a lower-stress environment could be apparent in a higher-stress context.

Elevated levels of corticosterone, the primary stress hormone in rodents, lead to upregulation of the HPA axis. CRH receptor 1, a key initiator of the stress response, mediates endocrine, autonomic, immune, and behavioural responses to stress (Oh et al., 2020). Both glucocorticoid and mineralocorticoid receptors modulate the stress response (Liu et al., 2020). Increased activation of these receptors can overstimulate the HPA axis, resulting in stress-induced anxiety. These aberrant expression levels, observed in stressed animals, were successfully reduced with *L. rhamnosus* administration. Lower corticosterone levels and reduced receptor activation suggest decreased HPA axis stimulation, leading to reduced stress responses and less pronounced stress-induced behavioural changes.

In avian models, SFP, a behavioural marker for anxiety-like behaviour (Mott et al., 2022), is regulated by serotonin and dopamine (van Hierden et al., 2002, 2004; Kjaer and Guémené, 2009; Kops et al., 2013; Birkl et al., 2017; Birkl et al., 2019). Serum levels of these neurotransmitters are regulated by their precursors, such as tryptophan, tyrosine, and phenylalanine (Fernstrom and Fernstrom, 2007; Mindus et al., 2021b). For example, lower tryptophan levels result in reduced serotonin bioavailability, both of which have been observed in stressed animals. *L. rhamnosus* treatment normalised these levels. The reduced serotonin and dopamine levels are further corroborated by the elevated levels of their metabolites, 5-HIAA and DOPAC, reported in stressed animals. Increased levels of these metabolites indicate heightened neurotransmitter breakdown (Zetterström et al., 1988), highlighting the biochemical imbalances caused by stress.

Stressed animals typically exhibit lower expression levels of GABA and alterations in GABA_A_ and GABA_B_ receptor expression. *L. rhamnosus* has been shown to increase GABA expression and modulate changes in GABA_A_ and GABA_B_ receptor expression. Previous research has reported a significant rise in brain GABA levels following *L. rhamnosus* consumption (Janik et al., 2016; Faucher et al., 2022). GABA_B_ receptors, crucial in mood and anxiety disorders, were found to have reduced mRNA levels in stressed animals, which *L. rhamnosus* treatment effectively increased. Studies on anxiety and depression have consistently noted decreased expression of GABA_B_ receptors (Felice et al., 2022). Moreover, the role of GABA_A_ receptor subunits in benzodiazepine-mediated effects underscores their significance in anxiety modulation, with the α2 subunit implicated in anxiolysis (Rudolph and Möhler, 2004). Animals treated with *L. rhamnosus* and exhibiting higher hippocampal GABA_A_α2 mRNA levels correlated with reduced anxiety levels (Bravo et al., 2011).

Glutamatergic signalling is pivotal in transmitting stress-related information within the hypothalamic–pituitary–adrenal (HPA) axis. Dysregulation in glutamatergic neurotransmission has been linked to HPA axis dysfunction and anxiety disorders (Kinlein et al., 2022). The reduced levels of glutamine and glutathione observed in stressed animals suggest a disruption in the GABA/glutamate cycle, where glutamine depletion may lead to downregulation of glutamate, a crucial precursor of GABA synthesis (Hasler et al., 2019; Kochalska et al., 2020). *L. rhamnosus* administration normalised these neurotransmitter and substrate levels in stressed animals, concurrently exerting anxiolytic effects (Kochalska et al., 2020).

Treated animals also exhibited elevated expression levels of G-protein-coupled receptor 43 (GPR43), which are activated by short-chain fatty acids and protect against oxidative stress-induced neuronal injury (Saikachain et al., 2023). Additionally, *L. rhamnosus* significantly increased metabotropic glutamate receptor 4 (GRM4) expression, implicated in mood disorders for its role in modulating neurotransmitter release and post-synaptic glutamatergic signalling (Chaki et al., 2019; Dalziel et al., 2023). Furthermore, the higher expression levels of total N-acetylaspartate (tNAA) induced by *L. rhamnosus* treatment suggest a neuroprotective mechanism against neuronal density loss, neuronal dysfunction, and mitochondrial dysfunction commonly observed in neurodegenerative diseases (Hemanth Kumar et al., 2012; Sturrock et al., 2015; Wang et al., 2015; Xu et al., 2016; Guan et al., 2017). Mitochondrial dysfunction, inferred from elevated pro-apoptotic protein levels in stressed animals, is implicated in stress-induced anxiety (Kochalska et al., 2020). *L. rhamnosus* mitigated these effects by normalising tNAA levels and modulating the apoptotic pathway, underscoring its neuroprotective potential (Kochalska et al., 2020). Histopathological improvements observed with *L. rhamnosus* treatment further support its role in reducing anxiety-related symptoms (Luykx et al., 2012).

Inflammation and immunomodulation are recognised contributors to stress and anxiety disorders (Morey et al., 2015; Réus et al., 2015; Hughes et al., 2016; Peirce and Alviña, 2019). Previous studies have reported decreased expression levels of TREG cells in conditions of chronic stress, anxiety, and post-traumatic stress disorder (Sommershof et al., 2009; Kim et al., 2012; Lindqvist et al., 2014). Conversely, heightened expression levels of peripheral monocytes have been associated with anxiety-like behaviour (Wohleb et al., 2013; Weber et al., 2017). These activated monocytes facilitate neuroinflammation by migrating to the brain, thereby exacerbating stress-induced anxiety. Lower microglial expression has been correlated with reduced anxiety levels (Li et al., 2021), whereas increased activation of Ly6C^hi^ monocytes and microglia in the hippocampus has been linked to heightened anxiety levels in stressed animals (Liu et al., 2020, 2021b). Administration of *L. rhamnosus* was found to mitigate these effects by reducing the proportion of activated microglia and alleviating anxiety-like behaviour in stressed animals, underscoring its anxiolytic properties. Treated animals also exhibited higher TREG cell levels than stressed animals, with these cells expressing anti-inflammatory cytokines such as IL-10. TREG cells play a critical role in maintaining immune system balance and peripheral tolerance and dampening excessive immune responses and autoimmunity (Mindus et al., 2021a). The observed increase in TREG cells following *L. rhamnosus* treatment may signify a response to the pro-inflammatory milieu observed in stressed animals. Moreover, *L. rhamnosus* treatment was associated with elevated BDNF levels. BDNF is a neuroprotective and anti-inflammatory factor and acts as a negative regulator of the HPA axis. By enhancing BDNF levels, *L. rhamnosus* may potentially mitigate HPA axis hyperactivity and consequent stress responses (Liu et al., 2020).

Stressed animals also exhibit elevated expression levels of pro-inflammatory cytokines compared to control and treated groups. LPS, a key marker of inflammation, triggers the activation of the TLR4/NF-κB signalling pathway, leading to increased production of pro-inflammatory mediators, including iNOS and COX-2 (Lu et al., 2008). LPS can also permeate the bloodstream, disrupting microvascular homoeostasis and blood–brain barrier integrity, thereby causing tight junction disorders and promoting the release of additional pro-inflammatory cytokines such as TNF-α (Blanchette and Daneman, 2015). Administration of *L. rhamnosus* has been demonstrated to significantly attenuate the levels of these pro-inflammatory markers in stressed animals (Huang et al., 2023). Moreover, *L. rhamnosus* treatment enhances the expression levels of tight junction proteins such as ZO-1 and occludin, which are essential for maintaining blood–brain barrier integrity (Huang et al., 2023). This dual action of *L. rhamnosus* in reducing neuroinflammation and bolstering barrier function highlights its therapeutic potential in mitigating stress-induced disruptions to immune and neurovascular systems.

Treatment with *L. rhamnosus* has been observed to effectively modulate the GM and ameliorate dysbiosis in stressed animals. Previous research has associated heightened levels of Proteobacteria and *Desulfovibrio* with anxiety-like behaviours (Jiang et al., 2015; Zhu et al., 2019; Rychlik, 2020), which were indeed found in stressed animals in our review. Conversely, animals treated with *L. rhamnosus* exhibited an increased abundance of taxa commonly associated with health in prior studies, such as *Lactobacillus* and *Faecalibacterium* (Khan et al., 2020). Moreover, *L. rhamnosus* treatment was shown to elevate butyric acid levels in stressed animals. Increasingly recognised for its role in the microbiota–gut–brain axis, butyric acid exerts multiple beneficial effects, including inhibition of intestinal pathogen adhesion, maintenance of intestinal and blood–brain barrier integrity, anti-inflammatory properties, and neuroactive effects (Argañaraz-Martínez et al., 2013; Li et al., 2016; Liu et al., 2021a; Miao et al., 2022; Yosi et al., 2022). Butyric acid has also been demonstrated to promote intestinal health by upregulating mRNA levels of ZO-1, enhancing transmembrane resistance of epithelial cells, and safeguarding intestinal barrier integrity (Xiao et al., 2023). Therefore, through modulation of the GM and enhancement of butyric acid levels, *L. rhamnosus* may indirectly mitigate factors contributing to stress-induced anxiety by protecting intestinal integrity, reducing inflammation, and promoting healthy microbiota profiles. These findings underscore the potential therapeutic implications of *L. rhamnosus* in managing stress-related disorders via modulation of the MGB axis.

## Limitations

The mechanistic pathways identified in this review should be interpreted as putative rather than definitive. Since each mechanism is supported by only a small subset of the included studies, these pathways likely represent preliminary mechanistic hypotheses rather than established causal routes. Discrepancies in research findings on the efficacy of *L. rhamnosus* in treating stress-induced anxiety may arise from various factors, including the use of different bacterial strains, variations in dosage, and the severity of anxiety among subjects. Different strains of *L. rhamnosus* may have varying effects on anxiety, leading to inconsistent results across studies. Additionally, the dosage and duration of treatment can significantly influence outcomes, with some studies possibly using suboptimal doses that do not elicit a therapeutic effect (Wright et al., 2011). The severity of anxiety among study subjects also plays a crucial role, as animals with mild anxiety may respond differently compared to those with more severe symptoms (Taylor et al., 2018). Future research should aim to standardise these parameters to mitigate these discrepancies and reduce bias. Employing consistent strains, dosages, and well-defined criteria for anxiety severity will enhance the reliability and comparability of study results, ultimately contributing to a more accurate understanding of the potential of *L. rhamnosus* as a treatment for anxiety. The variability in age and sex among animals in the included studies introduces a degree of biological heterogeneity that can influence our overall conclusions about the therapeutic effectiveness of *L. rhamnosus.* Age-related differences in neuroplasticity, metabolism, and immune response may lead to variations in therapeutic outcomes, while sex-based differences, particularly in hormone-regulated pathways, can further modulate treatment efficacy. Although there is a predominance of male animals in the studies, the inclusion of some female subjects allows for at least partial consideration of sex-specific responses. More notably, the presence of multiple species, such as rats, mice, and hens, demonstrates that the therapeutic effect of *L. rhamnosus* is not confined to a single model, supporting its broader potential applicability. To enhance the robustness of future studies, a more balanced representation of sexes and a more systematic comparison across species and age groups would be beneficial in refining our understanding of the general applicability of *L. rhamnosus.* Additionally, despite promising results in animal studies, there is a notable lack of human studies examining the efficacy of *L. rhamnosus* in reducing stress-induced anxiety, as preliminary searches revealed only a limited number of relevant studies. During the initial screening phase, 29 articles were identified as human studies. Of these, 25 primarily investigated anxiety related to factors such as trauma, abuse, and depression, rather than stress-induced anxiety, leading to their exclusion. Only four studies specifically examined stress-induced anxiety; however, these studies also presented notable limitations. Several of these studies took place during the COVID-19 pandemic, which hindered follow-up with all participants and led to uncertainties regarding adherence to daily capsule intake due to the lack of in-person appointments (Slykerman et al., 2022; Slykerman and Li, 2022). Unlike animal studies, where *L. rhamnosus* was administered at consistent doses and fixed time points, it was not possible to confirm whether participants in the clinical studies consistently adhered to the prescribed treatment regimen. Furthermore, the impact of the COVID-19 pandemic on participants’ health introduced an additional confounding factor, as illness-related immune system alterations may have influenced study outcomes. This is particularly relevant given that animal studies have demonstrated a role of *L. rhamnosus* in modulating immune function. Notably, the pandemic was a period characterised by widespread psychological distress. While participant dropout is expected in clinical trials, attrition rates were particularly elevated during the pandemic. Additionally, healthcare staff shortages further hindered effective follow-up with participants, potentially impacting study outcomes and data reliability. Furthermore, these studies did not measure biochemical markers associated with stress-induced anxiety. While behavioural studies provide valuable insights, understanding the specific underlying pathways influenced by *L. rhamnosus* is essential for a comprehensive assessment of its therapeutic potential. The ability to evaluate its efficacy is limited in the absence of sufficient biological parameters that allow for direct measurement of its physiological and molecular effects. Although some studies reported both psychological and physiological improvements in stress and anxiety levels following treatment with *L. rhamnosus* (Zheng et al., 2021; Wauters et al., 2022), the small sample sizes necessitate further investigation. For instance, one study included only six participants, representing a highly limited sample size. While the findings were of interest, the small cohort significantly restricted the statistical power of the study. A larger sample size is necessary to ensure robustness, generalisability, and statistical significance of the results. This gap in research underscores the need for well-designed clinical trials to validate these findings in human populations. Understanding the specific impacts of *L. rhamnosus* on human anxiety will be crucial for translating these benefits into clinical practice.

## Conclusion

There is preclinical evidence of the anxiolytic effects of *L. rhamnosus* strains in animal models of stress-induced anxiety. *L. rhamnosus* strains were associated with changes in immunomodulation, anti-inflammatory effects, HPA axis regulation, neurotransmitter metabolism, and gut microbiome protection. However, these findings remain preliminary, and caution is warranted when translating them to humans. Well-designed, strain-specific clinical trials incorporating relevant biomarkers are needed to confirm efficacy, elucidate underlying mechanisms, and establish optimal dosing for anxiety management.

## Supporting information

10.1017/gmb.2025.10015.sm001 Juvale and Arulsamy supplementary material 1 Juvale and Arulsamy supplementary material

10.1017/gmb.2025.10015.sm002 Juvale and Arulsamy supplementary material 2 Juvale and Arulsamy supplementary material

10.1017/gmb.2025.10015.sm003 Juvale and Arulsamy supplementary material 3 Juvale and Arulsamy supplementary material

## Supplementary material

The supplementary material for this article can be found at http://doi.org/10.1017/gmb.2025.10015.
