# Supplementary material for: The therapeutic effects of Lacticaseibacillus rhamnosus on stress-induced anxiety: a systematic review of evidence from animal studies
Source: Gut Microbiome (Camb). 2025 Dec 17;6:e20. doi: 10.1017/gmb.2025.10015 (PMC12766537; doi:10.1017/gmb.2025.10015)
Supplement: Juvale and Arulsamy supplementary material 2 — Juvale and Arulsamy supplementary material [file S2632289725100157sup002.docx]

**Supplementary figure legends:**

**Supplementary Figure 1:**  **Preferred Reporting Items for Systematic Reviews and Meta-Analyses (PRISMA) style flowchart on literature search and selection results.**

**Supplementary Figure 2:** **Risk of bias assessment using the Systematic Review Centre for Laboratory Animal Experimentation (SYRCLE)**
