# Supplementary figures and images for: The therapeutic effects of Lacticaseibacillus rhamnosus on stress-induced anxiety: a systematic review of evidence from animal studies
Source: Gut Microbiome (Camb). 2025 Dec 17;6:e20. doi: 10.1017/gmb.2025.10015 (PMC12766537; doi:10.1017/gmb.2025.10015)

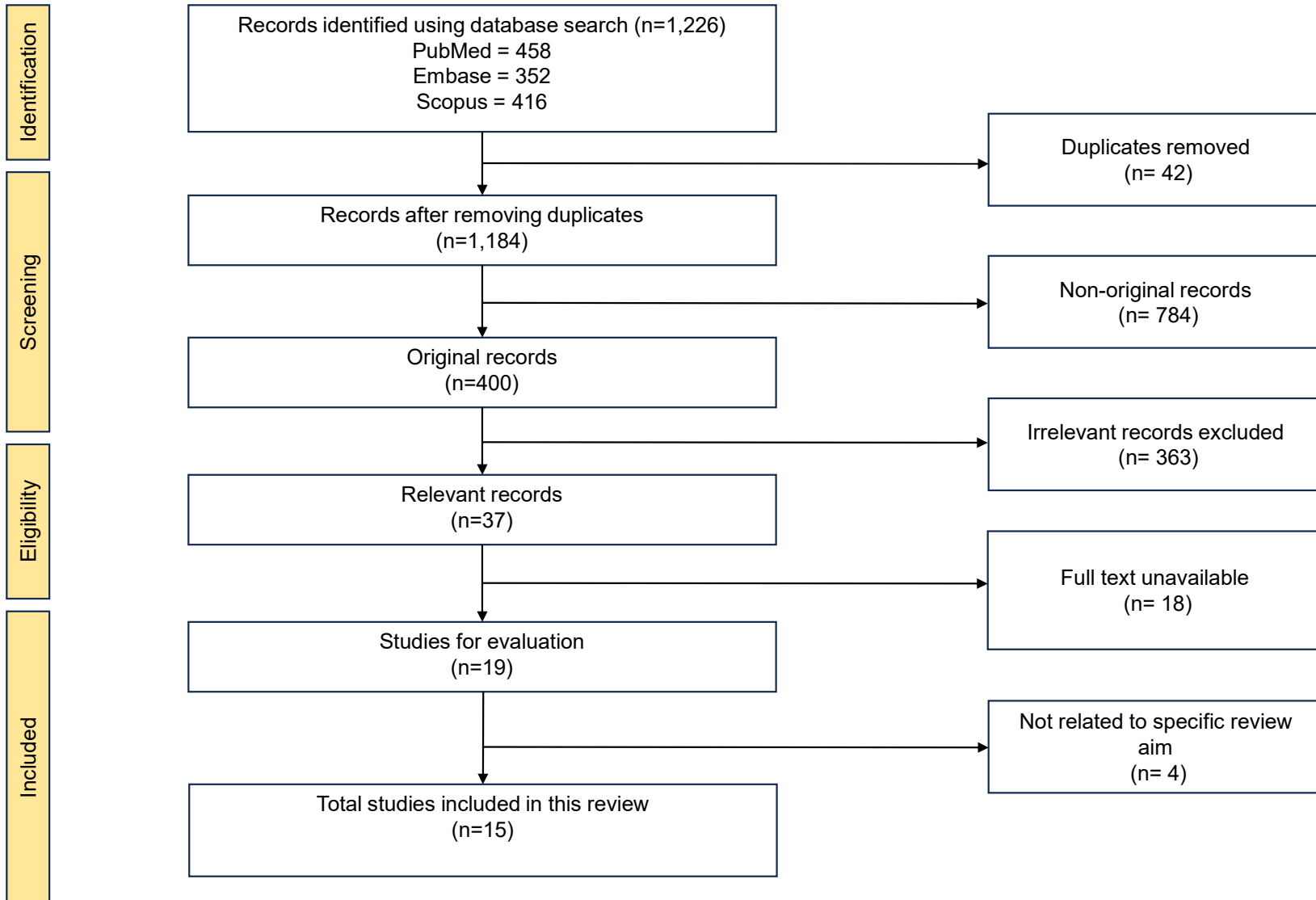

Supplement: Juvale and Arulsamy supplementary material 3 — Juvale and Arulsamy supplementary material [file S2632289725100157sup003.pdf]
